# Baby don't cry, genetic regulation of the weeping phenotype in *Prunus mume*

**DOI:** 10.1111/ppl.13229

**Published:** 2020-10-21

**Authors:** Sam W. van Es

**Affiliations:** ^1^ Department of Plant Physiology Umeå University Umeå Sweden

**Trees are an elegant and majestic lifeform and have for centuries been used by humans to create more pleasurable surroundings. One type of tree that perhaps sparks the imagination more than any other is one that exhibits the weeping branch phenotype. Recognized for their beauty, their drooping, downward facing branches makes them much sought after in parks and gardens. Despite a pretty clear idea of the structural difference of the weeping phenotype, little is known on the molecular regulation of the trait. In this issue of Physiologia Plantarum, Mao et al. (** **2020** **) screened for candidate genes related to the weeping phenotype to enhance our understanding of the molecular basis behind this characteristic way of growth.**

Though not typically found in nature, weeping trees have been cultivated for centuries for their elegance and adorn many a park and garden throughout the world. More than 500 weeping tree cultivars have been described of which some are grafted on the rootstock of a standard variety for support and others even need additional support without which they would grow as a weeping ground cover. This weeping trait is interesting from an architectural point of view but also deserves some attention from a biological point of view. In most cases stems grow upward, and during development obtain rigidity through secondary growth and the formation of tension wood on the upper side of the branch. This ultimately leads to structures strong enough to support their weight and defy gravity. In trees with the weeping trait the drooping branches have been attributed to alterations in wood formation such as faster elongation of cells than secondary (strengthening) growth, resulting in stems that lack the strength to support their weight and as a result start bending down (Hollender and Dardick 2015).

Contrary to the physiology of weeping trees, much less is known on the genetics behind and the molecular regulation of this trait. In peach (*Prunus persica*) an allele was found, appropriately called WEEP, that at least partially is responsible for the trait (Hollender et al. 2018). In a totally different species, crape myrtle (*Lagerstroemia*), it was shown that genes involved in the biosynthesis and signaling of the plant hormone gibberellic acid (GA) play a major role in regulating the weeping phenotype (Li et al. 2020). GA has been hypothesized to play a role in the weeping phenotype in several species through its involvement in wood formation (reviewed by Hollender and Dardick 2015).

In this issue of Physiologia Plantarum, Mao and colleagues take a close look at the weeping habit in *Prunus mume*, known commonly by several names including Mei, Japanese apricot and Japanese plum. By comparing differentially expressed genes and plant hormone levels between direct progeny of a weeping and an upright parent they aim to unravel part of the molecular network underlying the weeping trait (Fig. 1). What is interesting about their approach is that they take a detailed look at the lower (abaxial) and the upper (adaxial) side of a branch; as mentioned earlier, it is the tension wood on the adaxial side that contributes to a rigid and upright growing branch. The authors find a higher auxin (indole‐3‐acetic acid, IAA) content in the abaxial side of branches in upright progeny and a higher gibberellin (GA_3_) content in the adaxial side in weeping progeny with genes involved in the biosynthesis and signaling of these two hormones showing a similar pattern. The authors furthermore find two genes that encode key enzymes in lignin biosynthesis that where lower expressed in the weeping progeny. Less lignin production and a reduction in secondary growth via the interplay of IAA and GA_3_ might explain the lack of tensile strength in weeping branches in *P. mume*.

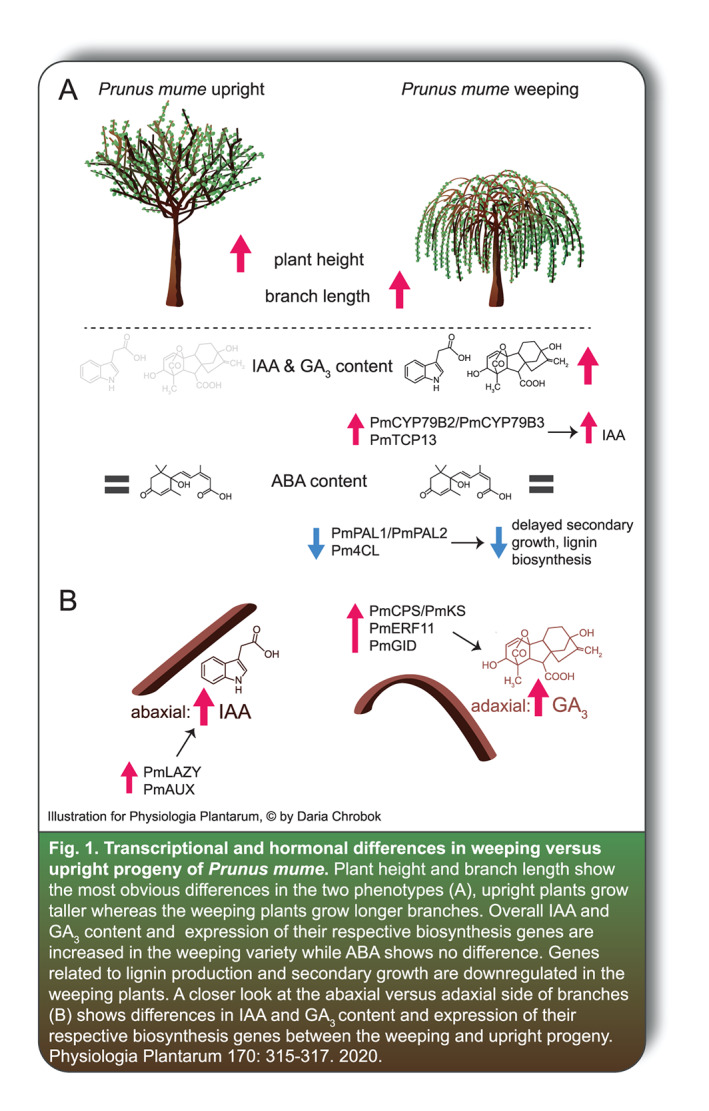

Rather than focusing on finding a single causal gene, the authors have provided a comprehensive overview of transcriptional and hormonal changes in an effort to reveal the differences between ‘upright’ and ‘weeping’ plants. They show that it is a complex trait with several hormones and molecular processes involved. Though much work is still to be done in order to unravel the regulation of the weeping trait, this study provides an excellent and interesting step, helping us to understand why some trees cry.
